# Lynn Sinclair Gillis, MB ChB, MD, FRCPsych

**DOI:** 10.1192/bjb.2020.126

**Published:** 2021-06

**Authors:** Joan Raphael-Leff

**Formerly Head of the Department of Psychiatry, University of Cape Town, and consultant psychiatrist, Groote Schuur Hospital, Cape Town, South Africa**

**Figure:**
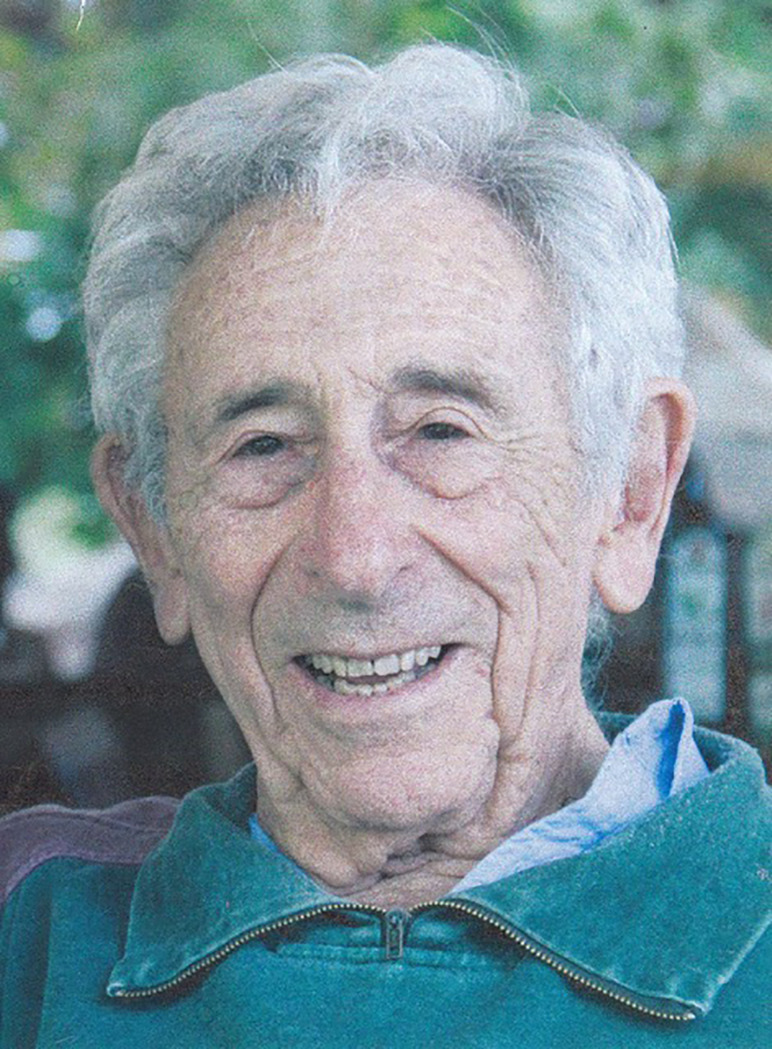

Lynn Gillis, who died recently at the age of 96 years, was, over a long period, one of the leaders of academic psychiatry in South Africa. In the 1960s and 1970s, he was a pioneer of community psychiatry and set up numerous community psychiatry services, mostly led by nurses. Under his guidance, a day hospital was established in 1963 in Cape Town, allied to a community service and psychiatric social club, which promoted continuity of care for patients in the community. This linked outreach provisions with psychiatric advocacy, aiming to destigmatise mental illness. Out-patient clinics were established at most hospitals and peripheral clinics in many parts of the country, which catered mostly for patients who had been discharged from hospital. Later legislation in 1976 made formal provision for a community service in country areas associated with particular psychiatric hospitals. Over time, an active Division of Child and Adolescent Psychiatry was established in Cape Town's Red Cross War Memorial Children's Hospital.

Courageously defying apartheid segregation, Lynn integrated staff across wards. In 1968, he carried out a significant research project into the rate of mental illness and alcoholism in the multiracial groups of people in the Cape Peninsula. Later, he organised a community service for alcoholism, and a specialised geriatric service, the first in South Africa. In 1980, he was appointed to head the newly established Social Psychiatry Medical Research Council Unit, which focused on research in community-based mental health. In collaboration with Professor Julian Leff of the UK Medical Research Council, he carried out a number of studies of the social precipitants of relapse in schizophrenia. Their studies revealed massive discrepancies in the lived experience of the different racial groups.

Lynn's clinical teaching and research laid the foundations for the existing Department of Psychiatry at the University of Cape Town, now a leader of psychiatric research on the African continent. Professor Dan Stein, the current Head of the Department, recalls that ‘the clinical, teaching, research, and social responsiveness strengths of the existing Department are in no small measure due to his pioneering work’. As well as clinical work and carrying out research, Lynn wrote several books dealing with different aspects of clinical practice in psychiatry and psychiatric education. Towards the end of his life he published a series of reflections on his rich and varied experience.

Lynn was born on 1 February 1924 to Jewish parents who had migrated from Kretinga in Lithuania. His father, Julius Gillis, was a dentist who grew competition roses as a hobby. His mother, Annie Gillis (née Lynn), was a concert pianist who gave music lessons locally. He was first brought up in the small South African town of Kroonstad in the Orange Free State. As a result, he spoke vernacular Afrikaans (a language he deemed second only to Yiddish in its rich array of metaphors and flamboyant curses) with fluency. At the age of 9 years, a year after the family had moved to Johannesburg, he contracted scarlet fever. His experience in the Children's Fever Hospital may have influenced his later ideas about hospitals as institutions.

He entered Witwatersrand medical school in 1941. He interrupted his medical studies to enlist as a medical assistant in the South African Medical Corps, serving in makeshift hospitals in North Africa and Italy. Returning to South Africa in 1945, he completed his medical studies, qualifying as a doctor in 1948. After qualification, he worked until 1962 at Tara Hospital, Johannesburg, a pioneering mental health facility where he was influenced by the indomitable Dr Mary Gordon, a migrant from Russia. In the 1950s, Lynn took a break from Tara to hold positions at both the Maudsley Hospital in London and St Francis Hospital, Haywards Heath in Sussex. In 1962, he was recruited to fill the position of founding Head of the Department of Psychiatry and Mental Health at the University of Cape Town and first consultant at Groote Schuur Hospital (posts he held for 27 years).

During his career, Lynn won numerous awards, among them the Salus Medal (silver) for Meritorious Service to Medicine (1989) and the Merit Award for Outstanding Services, Medical Association of South Africa (1990). He also held many positions of responsibility, including President of the South African National Council for Mental Health (1969–1970, 1976–1978, 1981–1983) and President of the College of Psychiatrists of South Africa (1969–1971). He was an elected member of the International Brain Research Organization (1977–1989), President of the South African Geriatrics Society (1978–1980), President of the South African Gerontological Association (1982–1993) and Chairman of the National Research Programme on Ageing of the South African Population, Human Sciences Research Council (1987–1991). He was a founding member and later Fellow of the Royal College of Psychiatrists (1971).

Although a reserved man, Lynn's warmth, compassion and mischievous humour influenced several generations of psychiatrists, psychologists and allied practitioners as much as his professional capacities as inspirational teacher, mentor and author of many publications. He had a long-lasting effect on his trainees, many of whom rose to eminence in South Africa, the USA and the UK. Today, they still acknowledge the lasting legacy of his singularly trusting style of leadership, which fostered personal initiative. Ever curious, his awareness of the many contradictions and unconscious processes of the human mind drew Lynn to psychoanalysis, and he pursued a lifelong interest in Buddhism. He always had a subtle appreciation of beauty, art and music. In retirement he studied sculpture and became a prolific creator of many austere carvings in marble and rare woods. An enthusiastic mountaineer, he remained remarkably healthy and agile until his last years. He was lucid and fiercely independent to the end of his full and fulfilled professional and artistic life. He died on 24 May 2020.

His wife Shirley (née Lurie) died in 2015 after they had been married for 64 years. One daughter, Susan, died in 2012. He leaves a daughter Jennifer, four grandchildren and three great grandchildren.

